# SOCIAL ISOLATION AMONG LGBT OLDER ADULTS: LESSONS LEARNED FROM A PILOT FRIENDLY-CALLER PROGRAM

**DOI:** 10.1093/geroni/igz038.2721

**Published:** 2019-11-08

**Authors:** Angie Perone, Berit Ingersoll-Dayton, Keisha Watkins-Dukhie

**Affiliations:** 1 University of Michigan, Ann Arbor, Ann Arbor, Michigan, United States; 2 SAGE Metro Detroit, Detroit, Michigan, United States

## Abstract

Lesbian, gay, bisexual, and transgender (LGBT) older adults face heightened risks of social isolation, given decades of discrimination. Research on telephone buddy programs with non-LGBT participants have proved predominantly unsuccessful at addressing social isolation and have found the greatest success with same-age matches. However, evidence suggests that LGBT adults may actually benefit from telephone buddy programs and in ways uniquely different from other groups. This article shares lessons learned from 30 participants across a 12-month pilot program that matched LGBT older adults to both LGBT and non-LGBT volunteer callers of various ages. One-third of participants identified as African American or Black. This project employed community-based participatory action research to identify, execute, and evaluate the program. Data includes information from questionnaires and telephone interviews prior to and during the program. In contrast to other research, data here revealed strong support for intergenerational matches. LGBT older adults of color especially benefited from program referrals and matches with/from LGBT adults of color, regardless of age. While the project aimed to capture two groups (LGBT older adults experiencing isolation and volunteer callers), the project revealed a third group: LGBT adults at risk of social isolation. This third group usually emerged among the “volunteer” callers but also identified concerns and risk factors for social isolation. The program also revealed unexpected benefits to both LGBT and non-LGBT volunteers, including less loneliness and a stronger sense of community. This article concludes with recommendations for developing similar programs to reduce social isolation in the LGBT community.

